# Synthesis of Sodium Alginate–Silver Nanocomposites Using Plasma Activated Water and Cold Atmospheric Plasma Treatment

**DOI:** 10.3390/nano11092306

**Published:** 2021-09-05

**Authors:** Nusrat Sharmin, Chengheng Pang, Izumi Sone, James Leon Walsh, Cecilia Górriz Fernández, Morten Sivertsvik, Estefanía Noriega Fernández

**Affiliations:** 1 Department of Food Safety and Quality, Nofima AS, Osloveien 1, 1430 Ås, Norway; 2 Department of Chemical and Environmental Engineering, University of Nottingham Ningbo China, Ningbo 315100, China; chengheng.pang@nottingham.edu.cn; 3 Department of Processing Technology, Nofima AS, Richard Johnsens Gate 4, 4021 Stavanger, Norway; Izumi.sone@nofima.no (I.S.); morten.sivertsvik@nofima.no (M.S.); estefania.noriegafernandez@efsa.europa.eu (E.N.F.); 4 Centre for Plasma Microbiology, Department of Electrical Engineering & Electronics, University of Liverpool, Liverpool L69 3BX, UK; J.L.Walsh@liverpool.ac.uk; 5 IES Escultor Juan de Villanueva, N-634, s/n, 33519 Pola de Siero, Spain; Cecilia.fernandez@nofima.no; 6 European Food Safety Authority, Via Carlo Magno 1A, 43126 Parma, Italy

**Keywords:** silver nanoparticles, plasma activated water, cold plasma, antimicrobial activity, mechanical properties

## Abstract

In this study, sodium alginate (SA)-based, eco-friendly nanocomposites films were synthesized for potential food packaging applications using silver nitrate (AgNO_3_) as the metal precursor, reactive nitrogen and oxygen species (RNOS) created within plasma activated water (PAW), or through cold plasma treatment (CP) as reducing agent and SA as stabilizing agent. The formation of silver nanoparticles (AgNPs) was confirmed via the absorption peaks observed between 440 and 450 nm in UV-vis spectroscopy. The tensile strength (TS) and tensile modulus (TM) of the nanocomposite films were significantly higher than those of the SA films. An increase in the TS was also observed as the AgNP concentration was increased from 1 to 5 mM. The storage modulus (G’) of the nanocomposite solution was higher than that of the SA solution. The synthesis of AgNPs resulted both in a higher solution viscosity and a more marked shear-thinning effect. The synthesized AgNPs showed antimicrobial activity against both Gram-positive (*Staphylococcus aureus*) and Gram-negative (*Escherichia coli*) bacteria. The AgNPs were spherical in shape with an average size of 22 nm.

## 1. Introduction

In recent years, nanotechnology has gained significant interest as an emerging science with a large amount of potential in different fields, such as biomedicine, cosmetics, imaging, cancer therapy, targeted drug delivery, and food packaging. Among different nanofiller materials, silver nanoparticles (AgNPs) have been explored extensively for different industrial applications due to their unique physicochemical, optical, catalytic, and antimicrobial properties, high specific surface area, and thermal stability [1,2,3]. AgNPs are considered to be a new class of antimicrobials, providing a new means to combat a wide range of bacterial pathogens. Due to their high specific surface area, AgNPs are more able to interact with the membranes of bacterial cells [4]. Several studies have reported that AgNPs can damage the cell membrane, leading to structural changes that make the bacterial cell more permeable [5,6]. However, this effect is highly influenced by the size, shape, and concentration of AgNPs. It has been reported that the reduction in the size of AgNPs can lead to an increased antibacterial activity [7,8]. Moreover, other functional properties of the AgNPs are also size- and shape-dependent [9]. Qin et al. reported the synthesis of quasi-spherical silver nanoparticles using ascorbic acid as the reducing agent and citrate as the stabilizing agent. The size of the silver nanoparticles produced varied between 73 and 31 nm, depending on the pH of the reaction system [10]. Rautela reported the synthesis of silver nanoparticles in the size range between 10 and 30 nm using tectona grandis seed extracts as the reducing agent [11]. Silver nanoparticles in the size range between 2 and 5 nm were synthesized extracellularly by the silver-tolerant yeast strain MKY3 [12]. Therefore, a significant amount of effort has been undertaken in the size-controlled synthesis of AgNPs.

Wet chemical synthesis of metal nanoparticles, which includes both the conventional chemical method and the novel biological method, has achieved significant success in recent years. Chemical reduction using different organic solvent, such as trisodium citrate, sodium borohydride, and ascorbate, is the most commonly used method for the synthesis of nanoparticles, which are often toxic and difficult to discard [10,13,14]. Moreover, the chemical reduction approaches require complicated purification methods [11]. This has led to the utilization of microorganisms, such as bacteria, yeast, fungi, and plant extracts, for the reduction of silver-to-silver nanoparticles, which excludes the requirement of a further purification step [11,12,15]. The major disadvantages of the use of a microbial source are the maintenance of aseptic conditions, the high cost of isolation, and their maintenance in culture media. However, one of the major challenges restricting the practical use of AgNPs is the production of nano-sized particles within a standard size range, in addition to a stable colloidal system to prevent agglomerations of the nanoparticles [16]. Therefore, there is an urgent need to use an eco-friendly reducing and stabilizing agent for the production of AgNPs.

Polysaccharides extracted from different plant sources may be a promising stabilizer for potential use with silver nanoparticles [16]. Alginate is a polysaccharide derived from seaweed, and is composed of β-D-mannuronic and α-L-guluronic acid residues linked by a β-(1-4) glycosidic bond [17]. Studies have shown that alginate can perform as a highly effective stabilizer, providing a high degree of stability against the aggregation for silver nanoparticles [16]. Another of alginate is that it has very good film-forming properties, which makes a highly suitable candidate for edible coating and food packaging applications. In addition, bio-nanocomposites prepared via the blending of different biopolymers with these nanofillers have been proven to increase the mechanical and gas barrier properties of the base polymer with extra functional properties such as ultraviolet light screening and antimicrobial properties [1].

Atmospheric plasma treatment is widely used for the chemical modification and surface treatment of polymeric materials due to its low cost, environmental sustainability, low energy consumption, and high efficiency [18]. Recently, the solution plasma process was proposed to be used in the production of metal nanoparticles and nanocomposites [19]. It has been reported that plasma created at atmospheric pressure and room temperature, i.e., cold plasma (CP), is abundant in energetic electrons capable of the dissociation, excitation, and ionization of gas molecules. Through these processes, CP is rich in energetic chemical species and photons with wavelengths extending from the infrared to the ultraviolet. The RONS in the CP discharge have been reported to diffuse/dissolve into the water during the exposure, and react with the water molecules, resulting in a cocktail of chemical species whose generation is subjected to the release of hydrogen ions [20]; for example, nitrites and nitrates are formed in the PAW through the dissolution of nitrogen oxides (NO_x_) formed in the air plasma by gas-phase reactions of dissociated N_2_ and O_2_. The dissolution of NO_x_ in water also produces H^+^ ions (drop in pH). Similarly, hydrogen peroxide is formed in the PAW through the recombination reaction of OH• radicals produced by plasma at the gas/liquid interface [20].

It has been widely reported that such an environment is suitable for silver reduction [21]. A recent application of CP technology is the production of plasma activated water (PAW) through the exposure of water to a CP discharge, e.g., using air as the working gas (creating air plasma), leading to the formation of a combination of reactive oxygen and nitrogen species (RONS) [22]. PAW is classified neither as a chemical reagent nor a natural resource, but as purified water, which contains a cocktail of reactive oxygen and nitrogen species [23].

The main aim of the current study was to develop a simple, chemical-free, and environmentally friendly method for the synthesis of alginate-silver nanocomposites with well-defined nanoparticle size using the cold plasma process and PAW. It was hypothesized that the reactive species present in PAW or produced during the plasma process will act as a reducing agent and reduce Ag^+^ from AgNO_3_ to AgNPs. Although alginate will act as a stabilizing agent for the AgNPs produced, it was also assumed that the AgNPs will have a good interaction with SA solution, which will result in enhanced mechanical, rheological, and antimicrobial properties. The physical properties of the nanocomposites were evaluated using UV-vis spectroscopy and transmission electron microscopy (TEM) equipped with an energy dispersive spectrometry (EDS). Alginate and nanocomposite films were formed using the solvent casting process, and the mechanical properties of the films were evaluated. The effect of different nanoparticle concentrations on the rheological and antimicrobial properties of the nanocomposites were also evaluated.

## 2. Materials and Methods

### 2.1. Materials

Sodium alginate (alginic acid sodium salt from brown algae) with guluronic acid content ~65–70% and mannuronic acid content ~5–35%, citric acid 99% (molecular weight: 192.12 g/mol) and nitric acid silver(I) salt ≥ 99.0% (molecular weight: 169.87 g/mol) were purchased from Sigma-Aldrich (Merck KGaA, Oslo, Norway).

### 2.2. Synthesis of Silver Nanoparticles

**Figure 1:**
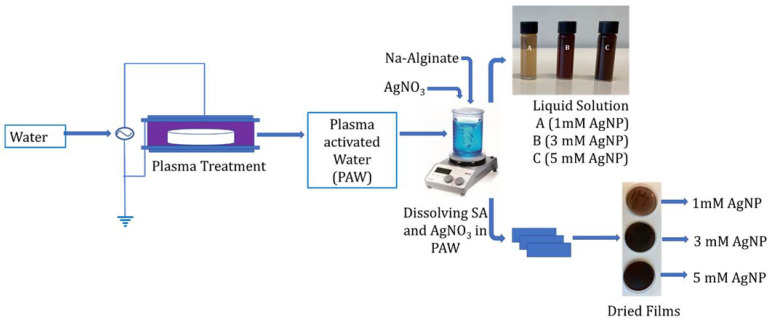
Schematic representation of the preparation of alginate-silver nanocomposites using plasma activated water.

The cold plasma (CP) system (developed inhouse in the Centre for Plasma Microbiology, Department of Electrical Engineering & Electronics, University of Liverpool) consisted of a powered and ground electrode adhered either side of a 1 mm thick quartz disc, forming a surface barrier discharge (SBD) system. The configuration was coupled to the lid of the treatment chamber with a dimension and total discharge area of 176 mm × 174 mm × 48 mm and 144 cm^2^, respectively. The system operated at atmospheric pressure, with room air as the working gas. For the treatment of 100 mL volume, the gap distance between the liquid surface and the electrode was 44.8 mm (3.2 mm water column). The plasma-generating source produced a sinusoidal voltage at a frequency of 18 kHz. An activation time of 30 min and plasma power of 36 W were selected for the generation of PAW from tap water. The generated PAW was used to produce 2% *w*/*v* sodium alginate solution containing 1, 3, or 5 mM AgNO_3_ for the synthesis of silver nanoparticles. The samples containing 1, 3, and 5 mM of AgNPs prepared using PAW were coded as PAW 1 mM, PAW 3 mM, and PAW 5 mM, respectively. Alternatively, 2% *w*/*v* sodium alginate containing 1, 3, or 5 mM AgNO_3_ was produced using tap water and then the same CP operating conditions were used to directly activate the sodium alginate–silver nitrate solution and coded as Plasma 1 mM, Plasma 3 mM, and Plasma 5 mM, respectively. The reactions were carried out in dark conditions in order to avoid the photo activation of AgNO_3_. The change in color from yellow to dark brown indicated the formation of AgNPs. The SA films were prepared by using a solution casting process. Films were cast by pouring 20 mL of the prepared solution into 90 mm diameter polystyrene petri dishes, which were allowed to dry for 24 h at room temperature. A flowchart of this process is shown in Figure 1.

The standard spectrophotometric method was used to determine the concentration of nitrates, nitrites, and hydrogen peroxide (see Table 1) present in the PAW and in tap water using a Shimadzu UVmini-1240-UV-VIS, Shimadzu, Tokyo, Japan. Concentration of nitrates was determined at 340 nm with the Spectroquant^®^ test kit #109713 (Merck, Oslo, Norway), analogous to DIN 38405-9. The Griess method (analogous to EPA 354.1, APHA 4500-NO2-B, and DIN EN 26 777, ANOVA, San Francisco, CA, USA) at 548 nm was used to quantify the nitrite levels [24]. The concentration of hydrogen peroxide was evaluated with the titanium sulphate colorimetric method at 407 nm [25]. The pH and oxygen reduction potential (ORP) values of PAW and TW were determined using a Mettler Toledo SevenGo Pro pH/ion meter (Mettler Toledo, Oslo, Norway).

### 2.3. UV-Visible Spectroscopy

The formation of AgNPs was confirmed by measuring the absorption spectra in UV–Visible spectroscopy (Shimadzu, UV-1280, Tokyo, Japan). The alginate solution with and without AgNPs was scanned at the speed of 300 nm min^−1^ in the 200–800 nm range.

### 2.4. Mechanical Properties

The mechanical properties of the films [Tensile strength (TS), tensile modulus (TM) and Elongation at Break (EB)] were measured using a TA.XT plus texture analyzer (Stable Micro Systems Ltd., Godalming, UK), following the ASTM D638-699 method (1999). Before the test, completely dried films were cut into the dimension of 60 mm × 15 mm × 0.02 mm (length × width × thickness), as recommended by the standard ISO 14125. The texture analyzer was equipped with a 500 kg load cell, with a crosshead speed of 1 mm/s, and the span distance was set at 25 mm. The thickness of the films was measured with a Digital Digimatic Vernier Caliper (Japan Mitutoyo 500-197-20/30 200 mm/ 8″; 0.01 mm resolution; ±0.02 mm accuracy, Tokyo, Japan). A minimum of three samples of each composition was tested and analyzed using the Exponent ver: 6.1.16.0 software (Microsoft, Oslo, Norway).

### 2.5. Rheological Properties

A hybrid rheometer (Discovery HR-2,TA Instruments, Newcastle, UK) was used to determine the rheological properties of the test solutions. The experiment was performed at a temperature of 22 °C with a cone and plate geometry (40 mm, 2°). Approximately 1 mL of nanocomposite solution was added onto the cross-hatched Peltier plate for each experiment. The plate was carefully washed with water and dried after each run. The frequency sweeps were conducted over the range of 0.1–150 rad/s (points per decade 5) at a constant strain of 1.0%. The shear dependency of the flow properties of the nanocomposites were determined over a shear rate range of 0.01–1000 s^−1^ (points per decade 5) with the maximum equilibration time of 60 s using the steady state sensing function in TRIOS software (TA Instruments, version 4.3, Oslo, Norway).

### 2.6. Antimicrobial Studies

The antimicrobial properties of the sodium alginate–silver nanocomposites were investigated using two typical Gram-negative and -positive bacterial strains, namely *Escherichia coli* (CCUG 10979) and *Staphylococcus aureus* (CCUG 1828), which were acquired from the Culture collection at the University of Gothenburg (Sweden).

Microbank™ beads of *E. coli* and *S. aureus* stored at −80 °C were spread onto Plate Count Agar (PCA; MERCK, Oslo, Norway) plates and incubated at 37 °C overnight. Then, a single colony was transferred into a 15 mL Falcon tube containing 5 mL of Tryptone Soya Broth (TSB; Oxoid) and incubated at 37 °C. After 24 h, appropriate serial decimal dilutions were prepared in TSB in order to achieve an initial cell concentration of 107 CFU/mL in the test solutions. To determine the initial levels of *E. coli* or *S. aureus* in the test solutions, appropriate decimal serial dilutions were prepared in saline solution (0.9% *w*/*v* NaCl; MERCK), plated onto Mueller Hinton Agar (MHA; MERCK) in triplicate and incubated at 37 °C for 24 h, prior to enumeration.

Either 10 (3.3% *v*/*v*) or 100 µL (33.3% *v*/*v*) of the test solutions was added to 1.5 mL Eppendorf tubes containing, respectively, 290 or 200 µL of the diluted *E. coli* or *S. aureus* cell suspensions in TSB. Control samples were prepared by adding 100 µL TSB to the bacterial suspensions. The Eppendorf tubes were incubated in a VorTemp 56 Shaking incubator (Labnet, Edison, NJ, USA) at 37 °C and 300 rpm. The antimicrobial trials were conducted at 37 °C, optimal for the growth of the tested bacterial strains, to simulate temperature abuse conditions. After 24 h incubation, appropriate decimal serial dilutions prepared in saline solution were then plated onto MHA plates in triplicate and incubated at 37 °C for 24 h, prior to enumeration. The antimicrobial assays were conducted at least in triplicate on independent days.

### 2.7. Transmission Electron Microscopy

A transmission electron microscopy (TEM) instrument equipped with energy-dispersive X-ray spectroscopy (EDS) was used to analyze the size distribution, shape, and dispersion of the AgNPs within alginate-silver nanocomposites. For the analysis, approximately 20 μL alginate-silver nanocomposite solution was pipetted on a 200 mesh copper grid followed by drying in a vacuum desiccator. Once completely dried, the image of the nanoparticles was obtained using a JEOL (JEM-2000FXII, JEOL, Birmingham, UK) TEM instrument operating at an acceleration voltage of 120 kV. The size distribution and average particle size of the AgNPs were determined via fitting the Lognormal Distribution Function in the particle size of TEM micrograph (Histogram Plot) using Image J (LOCI, University of Wisconsin, Madison, WI, USA).

### 2.8. Statistical Analysis

The statistical analysis was performed using the Prism software package (version 9.2, GraphPad Software, San Diego, CA, USA, http://www.graphpad.com, accessed on 10 June 2021). The two-way analysis of variance (ANOVA, San Francisco, CA, USA) was performed with the Bonferroni post-test to compare the significance of the change in one factor with time. The error bars represent the standard deviation with *n* = 3.

## 3. Results and Discussion

### 3.1. Color Change and UV-Visible Spectra

**Figure 2:**
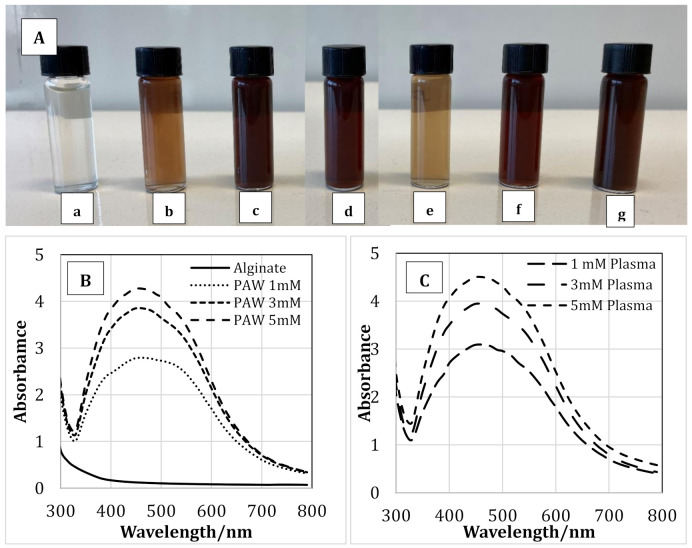
The appearance (color) of alginate solution (**A**-**a**), alginate-silver nanocomposites prepared using plasma activated water containing 1 (**A**-**b**), 3 (**A-c**) & 5 (**A-d**) mM silver nanoparticles and alginate-silver nanocomposites prepared using plasma treatment 1 (**A-e**), 3 (**A-f**) & 5 (**A-g**) mM silver nanoparticles. UV-Vis spectra of pure alginate and alginate containing 1, 3 & 5mM silver nanoparticles prepared using plasma activate water (**B**) and cold plasma (**C**).

**Figure 3:**
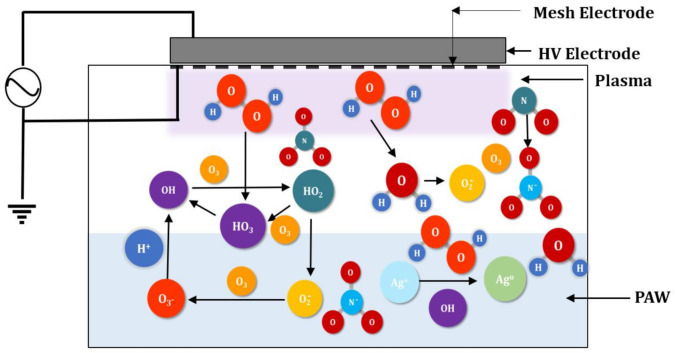
Schematic representation of the possible reduction reaction of silver ions into silver nanoparticles via the reactive species present in PAW. (Images are not drawn to the scale).

Figure 2A(a–g) shows the color of the pure alginate solution and the alginate-AgNP nanocomposites. As seen from the figure, the transparent colorless pure alginate solution changed its color to the characteristic pale orange and dark brown colors of silver nanoparticles. The reduction of Ag+ to Ago is responsible for the color change of the solution due to the excitation of surface plasmon resonance in AgNPs [26]. The intensity of the color depended on the concentration of AgNP produced. This change in color is an indication of the silver nanoparticle formation due to the change in plasmon resonance of silver nitrate because of the reduction process [27,28]. The solution containing 1 mM of AgNP showed a lighter color compared to the solutions containing 3 and 5 mM of AgNP. However, no difference in color was observed between the solutions containing AgNP prepared via PAW or plasma treatment. Therefore, it may be concluded that the RNOS present in PAW or created during the cold plasma treatment reduced the Ag^+^ to Ag^0^, and thus the color of the solution changed. Table 1 shows the concentration of the nitrates, nitrites, and hydrogen per oxide present in PAW. A schematic representation of a possible reduction phenomenon of Ag^+^ to Ag^0^ through plasma treatment is presented in Figure 3, and Equations (1)–(4) represent some of the reactions that take place during the plasma treatment:

(1) $${\mathrm{NO}}_{2}+O_{3}\to {\;\mathrm{NO}}_{3}+O_{2}$$

(2) $${\mathrm{NO}}_{3}+H_{2}O_{2}\;\to {\mathrm{HO}}_{2}+{\;H}^{+}+{\mathrm{NO}}_{3}^{-}$$

(3) $${\mathrm{NO}}_{3}+{\;\mathrm{NO}}_{2}^{-}\;\to {\mathrm{NO}}_{2}+{\;\mathrm{NO}}_{3}^{-}\;$$

(4) AgNO3+12H2O2→Ag0+12O2+HNO3

Synthesis of AgNPs via PAW or plasma within alginate solution was analyzed using UV-visible spectroscopy. Figure 2B,C shows the UV spectra of pure alginate and alginate-silver nanocomposites synthesized using PAW and plasma, respectively. As seen from the figure, the intensity of the peaks was dependent on the concentration of AgNPs used. For the samples prepared with PAW at a AgNP concentration of 5 mM, the highest peak was observed at 450 nm. As the AgNP concentration was reduced to 3 and 1 mM, the peak with the highest intensity was observed at 447 and 444 nm, respectively. The highest peak for solutions containing 1, 3, and 5 mM of AgNPs prepared using plasma treatment was observed at 443, 447, and 450 nm, respectively. Therefore, a red shift in the UV-visible spectra was observed with increasing AgNO_3_ concentration, which suggested the formation of silver nanoparticles [29]. Moreover, the shift is consistent with the color change of the sodium alginate solution as AgNPs were formed (Figure 2A). According to the literature, the typical absorption bands for silver nanoparticles are observed in the region of 350–450 nm [27,29,30,31]. The results obtained in our study coincide well with the previously reported data.

### 3.2. Mechanical Properties

**Figure 4:**
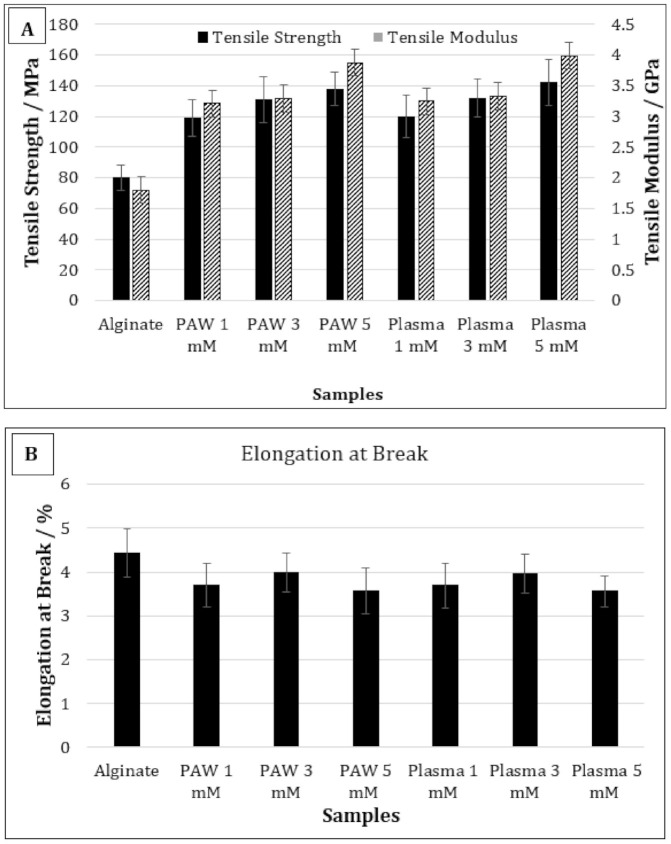
Tensile strength and tensile modulus (**A**) and elongation at break (**B**) of pure alginate and alginate-silver nanocomposites.

Figure 4A,B shows the TS, TM, and EB of pure alginate and alginate-silver nanocomposites containing 1, 3, and 5 mM of AgNPs prepared using PAW or plasma treatment. The TS of pure alginate was 80 MPa, which increased to 118 and 120 MPa for alginate-silver nanocomposites containing 1 mM of AgNP prepared using PAW and plasma treatment, respectively. No statistically significant difference was observed in the TS values as the AgNP content was increased from 1 to 3 mM. Moreover, no significant difference was observed between the TS values of the nanocomposites prepared using PAW and plasma treatment at any AgNP concentration. The TM values of pure alginate films were 1.8 GPa, which increased to ~3.2 GPa for nanocomposites containing 1 and 3 mM of AgNP using PAW or plasma. Therefore, composites containing 1 and 3 mM of AgNP prepared using PAW or plasma did not show any significant differences in their TM values. However, the TM of samples containing 5 mM of AgNP was significantly higher than the nanocomposites containing 1 and 3 mM of AgNP. Moreover, no statistically significant difference was observed between the 5 mM nanocomposites prepared with PAW or plasma treatment. On the contrary, addition of AgNP did not impart any significant effect on the EB of the alginate films at any concentration.

In general, the effect of metal or metallic nanoparticles on the mechanical properties of the polymer films depends on the compatibility between the base polymer and the nanofiller [2,32]. If the interaction between the nanofiller and the base polymer is stronger than that between the polymer chains, the mechanical properties will increase [32,33]. Therefore, a strong interaction between the nanofiller and the base polymer can positively affect the tensile strength and modulus. Shankar et al. studied the mechanical properties of silver-alginate nanocomposites and reported that the tensile strength increased by 16% compared to the alginate films when the nanocomposite films were prepared by reducing 1 mM AgNO_3_ with trisodium citrate as the reducing agent and polyvinyl pyrrolidine (PVP) as the capping agent [33]. Similar results were also observed by Kanagaraj et al. [34].

In our study, for films containing 1 mM of AgNP, the TS increased by approximately 33% compared to the control alginate films. The higher TS values observed in our study compared to those observed by Shankar et al. was due the fact that PAW or plasma was used as a reducing agent and the AgNPs were produced in situ within the polymer solution, resulting in better interaction [33]. It was reported in a previous study that the TS and TM values of the alginate films prepared using PAW were 33% and 26% higher than the alginate films prepared with deionized water, respectively [34,35]. The intersection of water and plasma can generate a cocktail of reactive oxygen and nitrogen species, with high oxidation redox potential and low pH [35]. Therefore, it was suggested that these active species can increase the crosslinking between the alginate chains, resulting in better mechanical performance of the films [35]. A possible crosslinking reaction between the alginate chains via the RNOS present in PAW has been reported in a previous publication [35]. In the present study, the TS and TM of films containing 1 mM of AgNP were 33% and 46% higher than the control alginate films, respectively. Therefore, although the improvement observed in TS values in the current study was similar to that observed with the film prepared with only PAW, the TM values were significantly higher, which indicates enhanced interaction between the AgNPs and alginate. Moreover, the TS and TM values of 5 mM AgNP composites were 14% and 16% higher than the 1 mM AgNP composites, respectively. Therefore, it may be concluded that not only PAW or plasma treatment, but also the presence of AgNP, had a significant effect on the improved TS and TM values of the nanocomposite films.

Gao et al. studied the effect of AgNP on the mechanical properties of cinnamaldehyde-polyvinyl alcohol nanocomposites, and reported that the TS increased but the EB remained unchanged with AgNP addition, as was observed in our current study [36]. They reported that the coating of aldehyde and the carboxyl group may reduce the attractive forces between the AgNPs, and thus increase the mobility of the nanoparticles, which can eventually result in increased mechanical properties. Shankar et al. reported that the presence of AgNP in alginate reduced the EB when added without any capping agent [33]. However, when the nanocomposites films were prepared in the presence of PVP as the capping agent, the EB increased. Therefore, it may also be concluded that the presence of RONS generated via PAW or plasma can potentially increase the mobility of AgNPs, resulting in improved mechanical properties.

### 3.3. Rheological Properties

**Figure 5:**
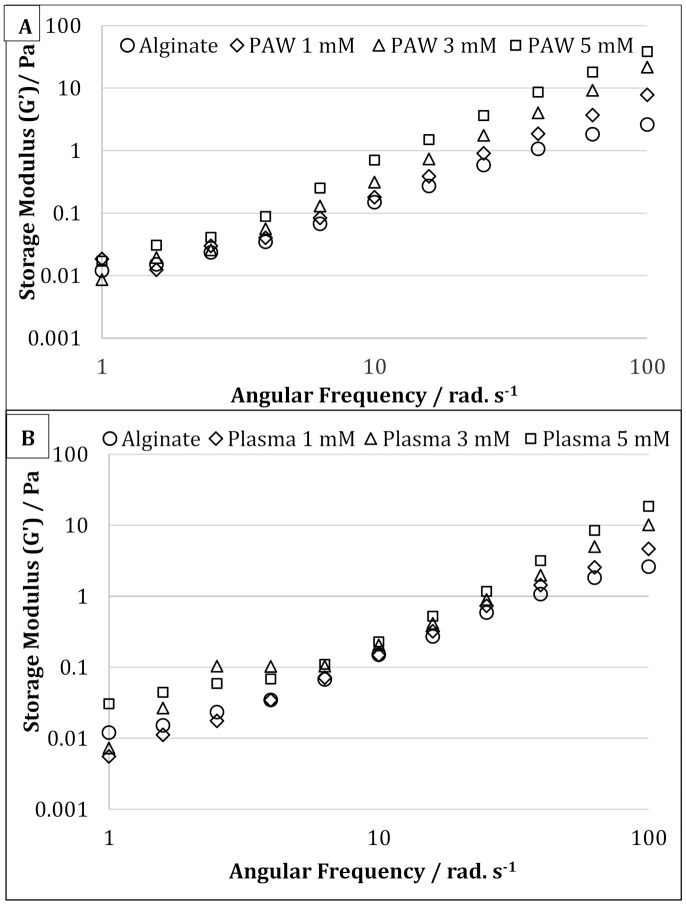
Oscillatory frequency sweep of pure alginate and alginate containing 1, 3, and 5 mM silver nanoparticles prepared using plasma activated water (**A**) and cold atmospheric plasma (**B**).

The storage modulus (G’) of the pure alginate samples and alginate-silver nanocomposites are shown in Figure 5A,B for AgNPs produced using PAW and plasma treatment, respectively. For the samples prepared using PAW, the G’ of the nanocomposites containing 5 mM of AgNPs were higher than that of the control sodium alginate solution at all frequencies. For samples containing 1 and 3 mM of AgNPs, the G’ at higher frequencies was higher than that of the sodium alginate solution (Figure 5A). However, at lower frequencies, samples containing sodium alginate, and 1 & 5 mM of AgNPs, showed similar G’ values. On the contrary, for the samples prepared with plasma, at lower frequencies (up to 2.5 rad s^−1^) and at higher frequencies (from 40 rad s^−1^ and above), the G’ increased in the following manner alginate < 1 mM < 3 Mm < 5 mM (Figure 5B). However, in the frequency range between 6 and 40 rad s^−1^, the samples showed similar G’ values. The increase in G’ is often attributed to an increase in the deformation energy, which may be due to the crosslinking between the alginate chains by AgNPs [35]. It has also been reported that the formation of crosslinks or supramolecular bonding between nanofiller and matrix can increase the shear strength between them, resulting in increased storage modulus and impact strength [37].

**Figure 6:**
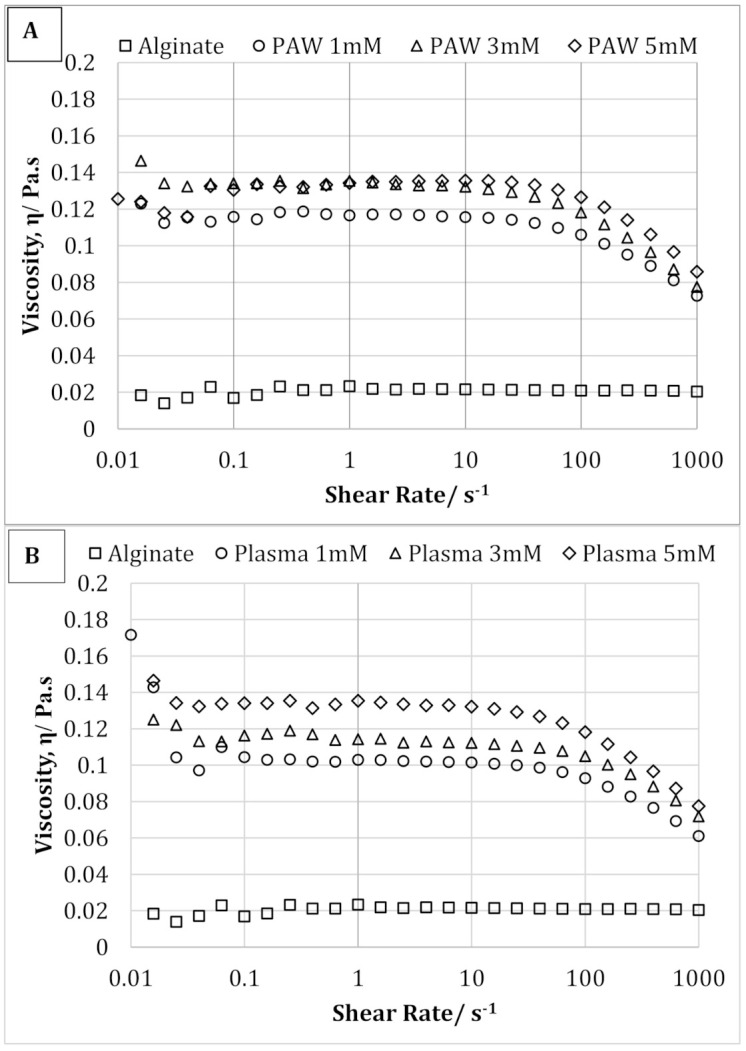
Shear flow curves of pure alginate and alginate containing 1, 3, and 5 mM silver nanoparticles prepared using plasma activated water (**A**) and cold plasma (**B**).

The effect of AgNP on the solution viscosity of sodium alginate solution is shown in Figure 6A,B. As clearly shown, synthesis of AgNPs resulted both in a higher solution viscosity and a more marked shear-thinning effect. Therefore, for AgNP-containing samples, a decrease in the viscosity was observed with increasing shear rate, which was not observed with pure sodium alginate solution. This behavior can be explained by the presence of entanglement, which increased with the addition of AgNPs and acts as a physical constraint, therefore significantly increasing the viscosity [38]. Moreover, the solution viscosity was also seen to increase with increasing AgNP concentration from 1 to 5 mM. However, increasing AgNP concentration did not show any significant effect on the shear-thinning behavior of the alginate-silver nanocomposites.

### 3.4. Antimicrobial Properties

**Figure 7:**
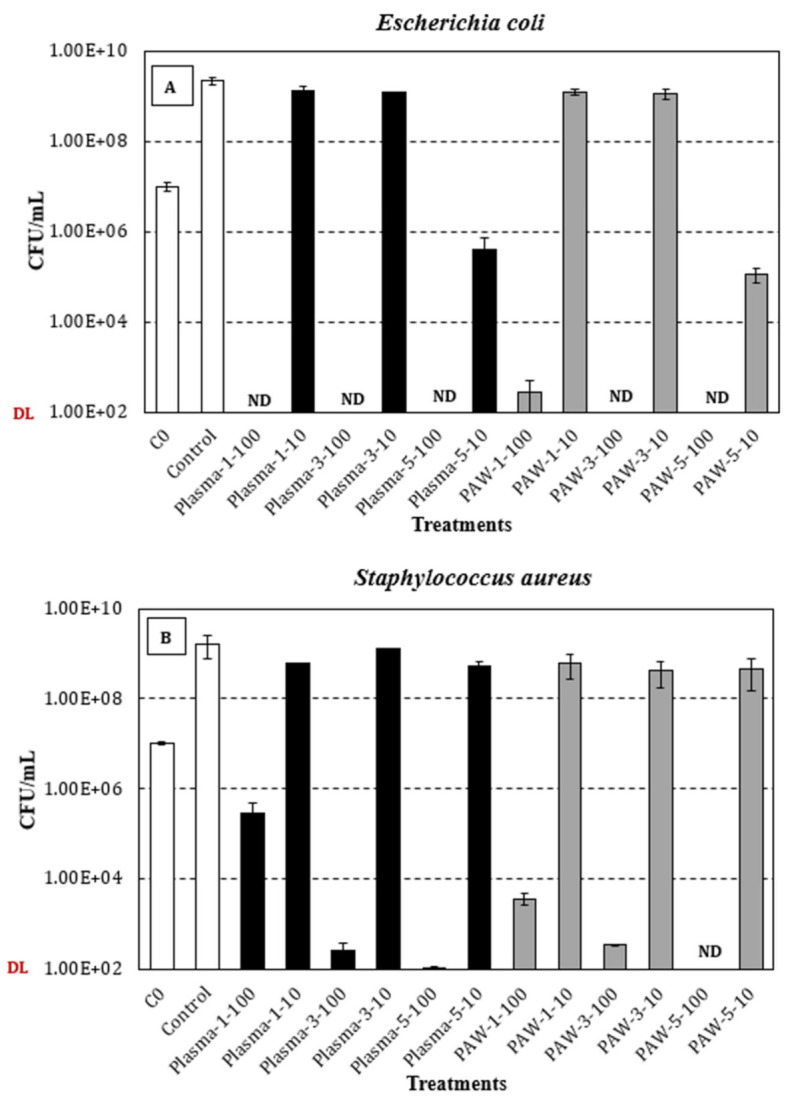
Viable counts (CFU/mL; average and standard deviation of at least three independent replicates) of *E. coli* (**A**) and *S. aureus* (**B**) after 24 h incubation at 300 rpm and 37 °C in samples containing either 10 µL (3.3% *v*/*v* in TSB) or 100 µL (33.3% *v*/*v* in TSB) of plasma-treated (black bars) or PAW-treated (grey bars) sodium alginate-AgNP nanocomposite-forming solutions. White bars: Initial concentration (C0) of *E. coli* (1.0 × 107 ± 2.4 × 106 CFU/mL) and *S. aureus* (1.0 × 107 ± 1.1 × 106 CFU/mL) in test/control samples, and bacterial levels in the control samples (TSB) after 24 h at 37 °C. ND: Not detected (detection limit = 102 CFU/mL).

Figure 7A,B shows, respectively, the concentration (viable counts, CFU/mL) of *Escherichia coli* and *Staphylococcus aureus* (average and standard deviation of at least three independent replicates) after 24 h incubation at 300 rpm and 37 °C in samples containing either 10 µL (3.3% *v*/*v* in TSB) or 100 µL (33.3% *v*/*v* in TSB) of sodium alginate-AgNP nanocomposite-forming solutions, and control samples (TSB). The antimicrobial trials were conducted at 37 °C, which represents temperature-abuse conditions, which are optimal for microbial growth.

Regarding the Gram-negative *E. coli* (Figure 7A), the average initial concentration for all the tested conditions was 1.0 × 107 ± 2.4 × 106 CFU/mL, and after 24 h at 300 rpm and 37 °C, stationary-phase levels of 2.3 × 109 ± 4.0 × 108 CFU/mL were achieved in the control samples. Overall, statistically significant differences were observed between the control and test solutions, regardless of the dose (10 or 100 µL), treatment (plasma or PAW), and silver nitrate concentration (1, 3, or 5 mM). Interestingly, the tested dose (10 or 100 µL) significantly affected the viability of *E. coli*, independently of the treatment (plasma or PAW) and silver nitrate concentration (1, 3, or 5 mM), typically resulting in no-detectable levels for the highest dose (100 µL) and concentrations ranging between 105 and 109 CFU/mL for the lowest dose (10 µL) of the test solutions. In general, significantly lower bacterial levels were achieved with increasing silver nitrate concentrations (1, 3, or 5 mM), irrespective of the treatment (plasma or PAW), with such a statistically significant difference being more noticeable for the highest silver nitrate concentration (5 mM), at the lowest assayed dose (10 µL). Wen-Fu Lee and Kai-Tai Tsao also reported a more pronounced inhibitory effect on the viability of *E. coli* cells with increased concentration of the AgNP [39]. Finally, when comparing the effect of plasma and PAW treatments, a similar trend was observed in terms of the viable count range, although the statistical analysis revealed significantly lower *E. coli* levels in PAW-treated solutions, except for the samples with the lowest silver nitrate concentration (1 mM) tested at the highest dose (100 µL). However, it is noteworthy that the *p*-value (0.0601) when comparing plasma and PAW treated samples at the above-mentioned condition was quite close to the significance level (0.05).

In relation to the Gram-positive *S. aureus* (Figure 7B), the average initial level (all conditions) and the maximum concentration at the stationary phase of growth in the control samples were, respectively, 1.0 × 107 ± 1.1 × 106 CFU/mL and 1.6 × 109 ± 8.9 × 108 CFU/mL. Similar to the results for *E. coli*, the control samples exhibited significantly higher viable counts than those achieved in the test solutions, irrespective of the different variables studied and the tested levels. Similarly, the tested dose (10 or 100 µL) significantly affected the viability of *S. aureus*, regardless of the treatment (plasma or PAW) and silver nitrate concentration (1, 3, or 5 mM), although the extent of this impact was not as pronounced as that reported for *E. coli.* Interestingly, significantly lower bacterial levels were observed with increasing silver nitrate concentrations (1, 3, or 5 mM), but only at the highest dose assayed (100 µL) of both plasma and PAW-treated solutions. For the samples containing 10 µL of the PAW-treated solutions, no significant differences in viable counts were observed with increasing silver nitrate concentrations. In the case of plasma-treated solutions (10 µL), similar results were observed, in terms of the viable count range, for the lowest (1 mM) and highest (5 mM) concentrations of silver nitrate, with a significant increase being noted at the intermediate concentration (3 mM). With regards to the effect of plasma and PAW, significant differences were observed for all the conditions assayed, except for the lowest silver nitrate concentration (1 mM) at the lowest dose (10 µL). Otherwise, significantly lower viable counts were achieved in the PAW-treated samples, excluding the samples containing 100 µL of the 3 mM silver nitrate solution.

To the best of the knowledge of the authors, antimicrobial properties inherent to sodium alginate have not been reported in literature. Thus, the observed inhibitory effect of the nanocomposites has been attributed to the presence of AgNPs. AgNPs have been reported to attach to the bacterial cell wall by electrostatic attraction and disrupt cell permeability and respiratory activity due to the generation of reactive oxygen species [28]. AgNPs bind with the thiol groups of DNA and RNA, which eventually affects bacterial protein synthesis [28,34,40]. AgNPs can also form pits on the cell surface, which can cause proton leakage and, eventually, cell death [41]. Overall, it is noteworthy that the inhibitory effect of the test solutions was more pronounced in *E. coli* than in *S. aureus*, which has been attributed to the different composition and structure of the respective cell walls. Indeed, Gram-negative bacteria are surrounded by a thin peptidoglycan cell wall, which itself is surrounded by an outer membrane containing lipopolysaccharides [42]. Gram-positive bacteria lack an outer membrane but are surrounded by layers of peptidoglycan thicker than those found in Gram-negatives [42]. AgNPs have been reported to significantly affect cell membrane integrity in Gram-negative bacteria (*E. coli* and *S. Typhimurium*), with no cell wall disruption being observed in Gram-positive bacteria (*S. aureus* and *B. subtilis*). However, sublethal AgNP levels caused cell membrane depolarization, leading to increased cell permeability, irrespective of the different cell wall composition in Gram-positive and -negative bacteria [43].

### 3.5. TEM and EDS Analysis

**Figure 8:**
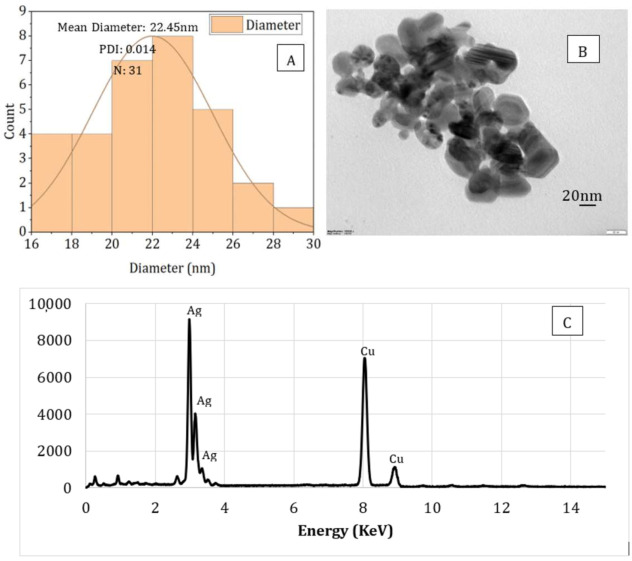
Particle size analysis (**A**), TEM image (**B**), and EDS (**C**) spectrum of sodium alginate-silver nanocomposites prepared using plasma activated water.

**Figure 9:**
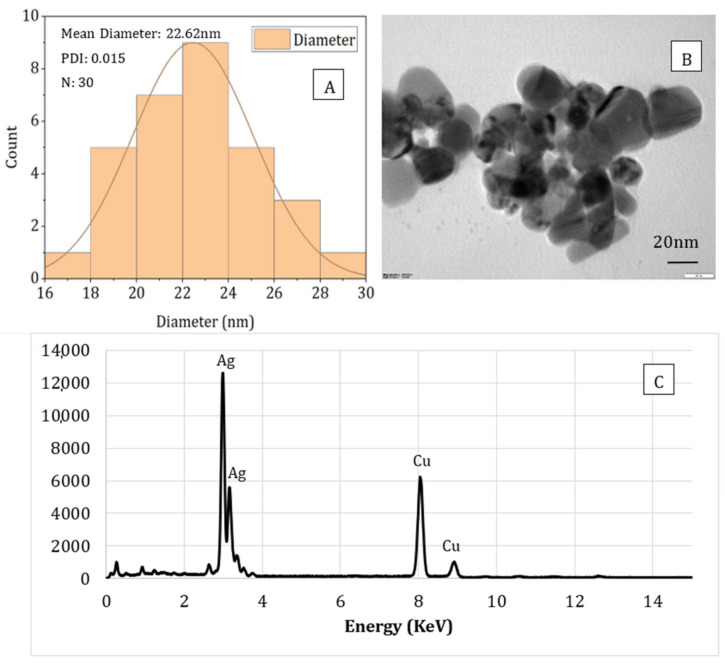
Particle size analysis (**A**), TEM image (**B**), and EDS spectrum (**C**) of sodium alginate-silver nanocomposites prepared using direct plasma treatment.

The particle size, shape, and compositional analysis of the AgNPs prepared were assessed using TEM and EDS mapping (Figure 8 and Figure 9). As seen from the figures for both processes (PAW and plasma), well-dispersed and spherical-shaped AgNPs were formed, which is in good agreement with UV-vis spectra and color change patterns on the samples. By photo processing using nano-measure software, the average particle size and particle size distribution of the AgNPs synthesized was analyzed. For both, similar particle size distributions were observed, with an average particle size of 22 nm. In addition, the EDS mapping displayed absorption peaks at 2.98, 3.18, and 3.38 keV (Figure 6) for the AgNPs produced using PAW, confirming the presence of elemental silver in AgNPs [44]. Similarly, the absorption peaks observed at 2.98 and 3.18 keV (Figure 7) for the samples prepared using plasma confirmed the presence of elemental silver in AgNPs. Moreover, the Cu peak observed for both samples was related to the conducting metallic surface coating. In addition, some small peaks were also observed for O, Na, Mg, and Sr (not indicated in the figure), which was related to the substrate.

## 4. Conclusions

In the present study, AgNPs with an average size of 22 nm were successfully synthesized using the reactive nitrogen and oxygen species present in the PAW, or produced during cold plasma treatment as the reducing agent within SA solution, which also acts as a stabilizing agent for the nanoparticles. Three different concentrations (1, 3, and 5 mM) of AgNO_3_ were used as metal precursor. The UV-vis spectroscopy and color change (from colorless alginate solution to light yellow and dark brown) of the solution confirmed the successful synthesis of AgNPs using both processes (PAW and cold plasma). The mechanical, rheological, and antimicrobial properties of the alginate-silver nanocomposites were also studied. For both processes (PAW and cold plasma), the tensile strength and modulus of the nanocomposite films were significantly higher than those of the SA films. The nanocomposite films containing 1 and 3 mM of AgNPs showed similar TS, whereas an increase in TS was observed when the AgNP concentration was increased to 5 mM. No statistically significant difference was observed between the EB of the SA and nanocomposite films. For nanocomposites prepared using PAW, the G’ was higher than that of the SA solution. An increase in G’ was also observed with increasing the nanoparticle concentration from 1 to 5 mM. For nanocomposites prepared using cold plasma treatment, the G’ of the nanocomposite solution was higher than that of the SA solution at high and low frequencies. The alginate-silver nano composites prepared using both processes (PAW and cold plasma) showed shear-thinning or non-Newtonian behavior, which was not observed in the SA samples. The antimicrobial properties of the sodium alginate–silver nanocomposites were investigated using two typical Gram-negative and -positive bacterial strains, namely, *Escherichia coli* and *Staphylococcus aureus*. Irrespective of the silver nitrate concentration and test dose, the nanocomposites prepared using both plasma and PAW treatments significantly inhibited the growth of both bacterial strains. However, PAW-treated nanocomposites, particularly at high silver nitrate concentrations and test doses, showed slightly better antimicrobial properties. In addition, a more pronounced inhibitory effect on the viability of *E. coli* cells was observed, compared to *S. aureus*, which was attributed to differences in cell wall structure and composition between Gram-negative and -positive bacteria. Nanocomposites containing 5 mM AgNPs showed the strongest inhibitory effect. The TEM images showed well-dispersed spherical-shaped AgNPs were formed with an average size of 22 nm from both processes (PAW and plasma). This study demonstrated that AgNPs can be effectively produced without harmful chemicals using PAW or plasma treatment and sodium alginate. The nanocomposites were successfully made into films and showed very good mechanical and antimicrobial properties for different alginate-based edible coating and food packaging applications.

## Figures and Tables

**Table 1:** Concentration of nitrates, nitrites, and hydrogen peroxide, pH, and ORP in plasma activated water (PAW) and the source tap water (TW).

	Nitrates (mg/L)	Nitrites (mg/L)	Hydrogen Peroxide (mg/L)	pH	ORP (mV)
TW	ND	ND	ND	8.0 ± 0.1	−45.3 ± 2.4
PAW	32.4 ± 5.6	462.3 ± 1.2	8.8 ± 0.4	2.3 ± 0.1	284.1 ± 11.5

## Data Availability

Not Applicable.

## Disclaimer

The author, Estefanía Noriega Fernández, is employed with the European Food Safety Authority (EFSA) at the Nutrition Unit that provides scientific and administrative support to the Panel on “Nutrition, Novel Foods and Food Allergens” in the area “Safety Assessment of Novel Foods”. However, the present article is published under the sole responsibility of the authors N. Sharmin, I. Sone, J. Walsh, M. Sivertsvik, and E. Noriega Fernández and may not be considered as an EFSA scientific output. The positions and opinions presented in this article are those of the author/s alone and are not intended to represent the views/any official position or scientific works of EFSA. For more information about the views or scientific outputs of EFSA, please consult its website under http://efsa.europa.eu (assessed on 15 June2021).
